# Abdominal wall hemorrhage after intravenous thrombolysis for acute ischemic stroke

**DOI:** 10.1186/1471-2377-13-6

**Published:** 2013-01-14

**Authors:** Se-A An, Jinkwon Kim, Sang Heum Kim, Won Chan Kim

**Affiliations:** 1 Department of Neurology, CHA Bundang Medical Center, CHA University, 59 Yatap-ro, Bundang-gu, Seongnam-si, Gyeonggi-do, 463-712, Korea; 2 Department of Radiology, CHA Bundang Medical Center, CHA University, 59 Yatap-ro, Bundang-gu, Seongnam-si, Gyeonggi-do, 463-712, Korea

**Keywords:** Abdominal wall hemorrhage, Extracranial hemorrhage, Thrombolysis

## Abstract

**Background:**

Thrombolysis is strongly recommended for patients with significant neurologic deficits secondary to acute ischemic stroke. Extracranial bleeding is a rare but major complication of thrombolysis.

**Case presentation:**

A 78-year-old woman presented with acute ischemic stroke caused by occlusion of the basilar artery. Clinical recovery was observed after successful recanalization by intravenous thrombolysis and intraarterial thrombectomy. However, the patient complained of sudden abdominal pain following the intervention and a newly developed abdominal wall mass was found. CT scan and selective angiography confirmed active bleeding from the left epigastric artery into the abdominal muscle layer and the bleeding was successfully managed by selective embolization of the bleeding artery.

**Conclusions:**

We report a rare case of abdominal wall hemorrhage after thrombolysis for acute ischemic stroke. The findings indicate that abdominal wall hemorrhage should be considered as a differential diagnosis in the presence of abdominal discomfort after thrombolysis for acute ischemic stroke.

## Background

Intravenous administration of tissue plasminogen activator (tPA) is an effective treatment for acute ischemic stroke, and, at present, tPA is the only approved thrombolytic agent which can be used within 4.5 hours of symptom onset [1,2]. One of major complications of thrombolytic therapy is intracranial or extracranial bleeding. Compared with intracranial bleeding, extracranial bleeding is a less frequently reported complication, but it could still be critical [3-7]. The incidence of severe extracranial bleeding has been variously reported (from less than 1% to 13%) [7-10]. Extracranial hemorrhage has been more frequently reported in patients with thrombolysis protocol violation [8]. Here, we report a rare case of a patient with abdominal wall hemorrhage caused by intravenous (IV) thrombolysis for acute ischemic stroke.

## Case presentation

A 78-year-old woman presented with an altered level of consciousness and sudden onset of right-sided weakness. She was stuporous and there was severe weakness of the right limbs (right upper limb weakness of grade 1 and right lower limb weakness of grade 1; National Institute of Health Stroke Scale (NIHSS) score, 16 points). The time from the onset of the symptoms to the arrival at our hospital was 90 minutes. She had cardiac surgery for atrial septal defect with pulmonary valve stenosis 40 years ago. She did not a medical history of hypertension, diabetes mellitus or dyslipidemia. Emergency computed tomography (CT) and CT angiography of brain confirmed an occlusion of the upper segment of the basilar artery (BA) and the right and left posterior cerebral arteries (PCAs) due to thrombus formation. Thrombolytic therapy with a standard dose of IV tPA (0.9 mg/kg body weight) was promptly commenced. However, there weren’t any clinical improvements in the patient’s condition until the end of tPA infusion; therefore, intra-arterial (IA) thrombectomy with a Penumbra catheter was performed. With a right femoral approach, a dose of unfractionated heparin (1500 units) was intravenously administered and cerebral angiography was performed; complete occlusion of the distal BA was observed. A Penumbra catheter with a Prowler108 catheter was inserted into the distal portion of the BA through the right vertebral artery. Mechanical thrombectomy via the Penumbra catheter was performed with manual suction. After successful recanalization with this procedure, the patient’s symptoms dramatically improved: the NIHSS score improved to 3 points.

**Figure 1:**
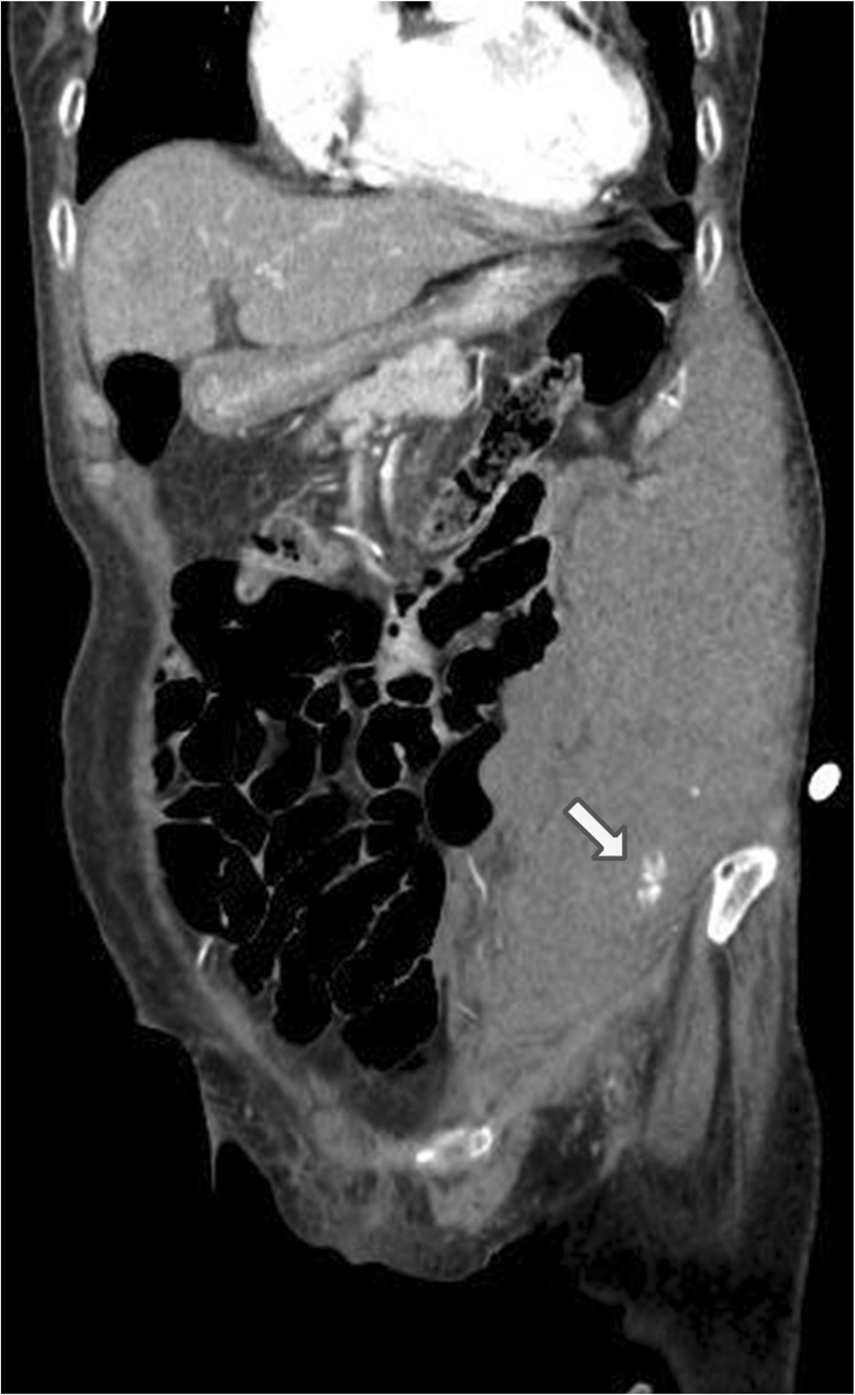
Abdominal CT with contrast showing a large hematoma and contrast extravasation (arrow) in abdomen.

**Figure 2:**
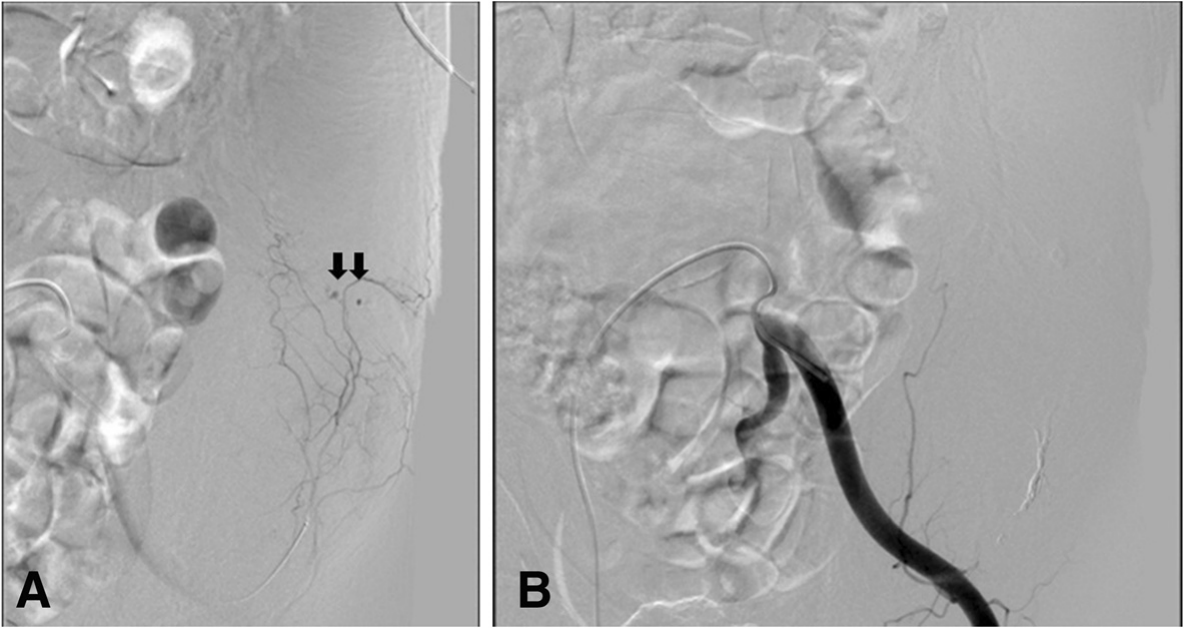
**Digital subtraction arteriogram of the left inferior epigastric artery. A**) Active bleeding is seen from several branches (arrows) of the inferior epigastric artery. **B**) The branches of the inferior epigastric artery embolized with polyvinyl alcohol.

However, after an hour, the patient suddenly complained of severe abdominal pain. Physical examination revealed the presence of an acutely enlarged mass on the left side of the abdominal wall. The patient’s blood pressure was 120/80 mmHg and the heart rate was 102/min. Blood tests revealed hemoglobin of 11.7 g/dL, hematocrit of 35.4% and platelet count of 205,000/mm^3^. Contrast-enhanced abdominal and pelvic CT image demonstrated a large intramuscular abdominal wall hematoma and active contrast extravasation within the hematoma, which was considered to be the focus of active bleeding (Figure 1). To prevent further bleeding, selective arteriography was performed and the left inferior epigastric artery was identified as the source of active bleeding. The proximal part of the bleeding artery was embolized with polyvinyl alcohol (250 μm), and the mixture of histoacryl and lipiodril (Figure 2). After the embolization, the patient stopped complaining of abdominal pain, and the abdominal wall hematoma stopped growing in size. During the rest of the hospitalization, her vital signs and hemoglobin level remained stable. On the 14th day, the patient was discharged home because she acquired a complete functional independence with only mild dysarthria (NIHSS score at the time of discharge was 1).

Vascular interventions such as IA thrombectomy can cause vascular rupture and bleeding. However, in this case, the IA procedure was performed via the right femoral artery, and the bleeding occurred from the left epigastric artery, which had originated from the left external iliac artery; therefore, it was likely to be related to the IV thrombolysis. Abdominal wall hematoma has been rarely reported in patients with deep vein thrombosis who underwent IV thrombolysis or long-term low-molecular-weight heparin therapy [11,12]. To the best of our knowledge, this is the first description of a case of active bleeding from the abdominal wall associated with thrombolysis for ischemic stroke.

## Conclusion

We report a rare case of abdominal wall hematoma, one of unusual complications of systemic thrombolysis for acute ischemic stroke. Neurologists, therefore, must consider abdominal pain in a patient after thrombolysis for acute ischemic stroke as a presenting symptom of active abdominal wall hemorrhage.

### Consent

Written informed consent was obtained from the patient for publication of this case report and any accompanying images. A copy of the written consent is available for review by the Editor-in-Chief of this journal.

## Abbreviations

BA: Basilar artery; CT: Computed tomography; IA: Intra-arterial; IV: Intravenous; NIHSS: National Institute of Health Stroke Scale; tPA: Tissue plasminogen activator.

## Competing interest

The authors have no financial conflicts of interest.

## Authors’ contributions

SAA carried out literature search and wrote the first draft. JK drafted and revised the manuscript. SHK performed the interventional procedure and helped to draft the manuscript. WCK conceived this case report and wrote the final manuscript. All authors read and approved the final manuscript.

## Funding sources

None

## Pre-publication history

The pre-publication history for this paper can be accessed here:

http://www.biomedcentral.com/1471-2377/13/6/prepub
